# Acid-base balance and hydration status following consumption of mineral-based alkaline bottled water

**DOI:** 10.1186/1550-2783-7-29

**Published:** 2010-09-13

**Authors:** Daniel P Heil

**Affiliations:** 1 Movement Science/Human Performance Laboratory, Department of Health & Human Development, H&PE Complex, Hoseaus Rm 121, Montana State University, Bozeman, MT USA

## Abstract

**Background:**

The present study sought to determine whether the consumption of a mineral-rich alkalizing (AK) bottled water could improve both acid-base balance and hydration status in young healthy adults under free-living conditions. The AK water contains a naturally high mineral content along with Alka-PlexLiquid™, a dissolved supplement that increases the mineral content and gives the water an alkalizing pH of 10.0.

**Methods:**

Thirty-eight subjects were matched by gender and self-reported physical activity (SRPA, hrs/week) and then split into Control (12 women, 7 men; Mean +/- SD: 23 +/- 2 yrs; 7.2 +/- 3.6 hrs/week SRPA) and Experimental (13 women, 6 men; 22 +/- 2 yrs; 6.4 +/- 4.0 hrs/week SRPA) groups. The Control group consumed non-mineralized placebo bottled water over a 4-week period while the Experimental group consumed the placebo water during the 1st and 4th weeks and the AK water during the middle 2-week treatment period. Fingertip blood and 24-hour urine samples were collected three times each week for subsequent measures of blood and urine osmolality and pH, as well as total urine volume. Dependent variables were analyzed using multivariate repeated measures ANOVA with post-hoc focused on evaluating changes over time within Control and Experimental groups (alpha = 0.05).

**Results:**

There were no significant changes in any of the dependent variables for the Control group. The Experimental group, however, showed significant increases in both the blood and urine pH (6.23 to 7.07 and 7.52 to 7.69, respectively), a decreased blood and increased urine osmolality, and a decreased urine output (2.51 to 2.05 L/day), all during the second week of the treatment period (P < 0.05). Further, these changes reversed for the Experimental group once subjects switched to the placebo water during the 4th week.

**Conclusions:**

Consumption of AK water was associated with improved acid-base balance (i.e., an alkalization of the blood and urine) and hydration status when consumed under free-living conditions. In contrast, subjects who consumed the placebo bottled water showed no changes over the same period of time. These results indicate that the habitual consumption of AK water may be a valuable nutritional vector for influencing both acid-base balance and hydration status in healthy adults.

## Background

Acid-base equilibrium within the body is tightly maintained through the interaction of three complementary mechanisms: Blood and tissue buffering systems (e.g., bicarbonate), the diffusion of carbon dioxide from the blood to the lungs via respiration, and the excretion of hydrogen ions from the blood to the urine by the kidneys. At any given time, acid-base balance is collectively influenced by cellular metabolism (e.g., exercise), dietary intake, as well as disease states known to influence either acid production (e.g., diabetic ketoacidosis) or excretion (e.g., renal failure). Chronic low-grade metabolic acidosis, a condition associated with "the Western diet" (i.e., high dietary intake of cheese, meats, and processed grains with relatively low intake of fruits and vegetables) has been linked with indicators of poor health or health risk such as an increased association with cardiometabolic risk factors [1], increased risk for the development of osteoporosis [2], loss of lean body mass in older adults [3], as well an increased risk for sudden death from myocardial infarction [4,5].

Given the evidence linking more acidic diets with increased risk for the development of chronic disease states, there is growing interest in using alkaline-based dietary interventions to reverse these associations. Several researchers have suggested, for instance, that mineral waters, especially those with high concentrations of calcium and bicarbonate, can impact acid-base balance [6] and contribute to the prevention of bone loss [7]. In fact, Burckhardt [7] has suggested that the purposeful consumption of mineral water represents one of the most practical means for increasing the nutritional alkali load to the body.

Recently, a highly mineralized glacier water, bottled together with a proprietary blend of mineral-based ingredients called Alka-PlexLiquid™ (Akali^®^; Glacier Water Compnay, LLC; Auburn, WA USA), was shown to rehydrate cyclists faster following a dehydrating bout of cycling exercise when compared with drinking non-mineralized bottled water [8]. This supplemented bottled water (hereafter referred to as AK) not only has a naturally high content of calcium, but the Alka-PlexLiquid™ supplement is purported to enhance both intracellular and extracellular buffering capacity as well as alkalizing the water to a pH of 10. This combination of high calcium content, a buffering agent, and alkalization may be functionally similar to the mineral waters described by Burckhardt [7] which suggests that bottled AK water could serve as a means for improving the body's nutritional alkali load with regular consumption. Recently, in fact, two studies have shown that the consumption of alkalizing nutrition supplements can have significant alkalizing effects on the body's acid-base balance using surrogate markers of urine and blood pH [9,10]. It is possible that the regular consumption of AK bottled water could have a similar influence on markers of acid-base balance, though this premise has not yet been evaluated in a controlled manner.

Given the previously demonstrated ability of AK water to rehydrate faster following a dehydrating bout of exercise, as well as the AK's potential influence as a dietary influence on acid-base balance, the present study was undertaken to systematically evaluate changes in both hydration and acid-base balance following chronic consumption of AK water in young healthy adults. Specifically, it was hypothesized that urine and blood pH, both common surrogate markers of whole body acid-base balance [11], would systematically increase as a result of daily consumption of the alkaline AK water. In addition, it was also hypothesized that the same chronic consumption of AK water could positively influence common markers of hydration status under free-living conditions. Thus, the potential influence of AK water on markers of both acid-base balance and hydration status were evaluated under free-living conditions with concomitant measures of both dietary intake and physical activity habits measured as potential covariates.

## Methods

### Subjects

College-aged volunteers (18-30 years) were recruited to participate in a multi-week evaluation involving the habitual consumption of bottled AK water under free-living conditions. Subjects read and signed an informed consent document approved by the Montana State University (MSU) Institutional Review Board (IRB) prior to testing. Subjects also completed a Health History Questionnaire that was used to screen out those with known chronic diseases or conditions known to influence acid production or excretion by the body. A self-reported physical activity (SRPA) questionnaire was administered prior to data collection to determine habitual levels of exercise, daily activities, or occupational-related activities that were performed at a moderate intensity or higher (i.e., ≥3 METS). Subjects were asked to maintain consistent weekly behaviors with respect to physical activity habits and dietary intake. In addition, subjects were asked to avoid the consumption of nutrition supplements with the exception of those that were taken on a daily basis (e.g., daily multivitamin). Data collection and sample processing, as well as subject meetings, all occurred in the Movement Science/Human Performance Lab on the MSU campus.

### Research Design and General Procedures

Prior to beginning a 4-week Testing Phase, subjects participated in a 3-day Pilot Phase during the preceding week with all subjects moving through both phases simultaneously. The 3-day Pilot Phase provided the opportunity to familiarize subjects with the requirements for data collection including the collection of bottled drinking water from the lab, the collection of 24-hour urine samples, the collection of early morning fingertip blood samples, the monitoring of free-living physical activity with a wrist-worn monitor, and the use of a diet diary. The goal of the Pilot Phase was to help ensure that subjects had enough training to effectively assist with their own data collection (e.g., 24-hour urine collection) during the Testing Phase.

Beginning the following Monday, the Testing Phase required four weeks of continuous data collection (Table 1). All subjects were assigned to drink non-mineralized bottled water (i.e., the placebo water) for the first (pre-treatment period) and fourth weeks (post-treatment period) of the Testing Phase to establish pre and post intervention baseline measures. For the second and third weeks of the Testing Phase (treatment period), however, the subject pool was split into two groups matched for SRPA and gender: The Control and Experimental groups. While the Control group continued to drink the same placebo water during the treatment period, the Experimental group drank the AK bottled water. Only the lead investigator was aware of which subjects were assigned to the Control and Experimental groups until the study's completion (i.e. Blind, Placebo-Controlled design).

**Table 1:** Four-week Testing Phase timeline for the consumption of bottled waters by Control and Experimental groups.

Week	Treatment Period	Control Group Water Consumed	Experimental Group Water Consumed
1	Pre-Treatment	Placebo Water	Placebo Water
2	Treatment	Placebo Water	AK Water
3	Treatment	Placebo Water	AK Water
4	Post-Treatment	Placebo Water	Placebo Water

Note: Placebo water was Aquafina while AK water was Akali^®^.

The daily data collection schedule was identical for each week of the Testing Phase (Table 2). Each day of the work week (Monday - Friday), as well as one day of the following weekend, subjects arrived at the lab early in the morning (6:30-8:30 AM) to provide a fingertip blood sample, or drop off their 24-hour urine collection containers, or both. Subjects were given the option of collecting their third weekly 24-hour urine sample on either day of the weekend that best allowed for such collection. This particular schedule was chosen to allow for the measurement of changes in both blood and urine pH and osmolality as each week progressed, as well as to accommodate the busy schedules of the student-volunteers. Additionally, body height and mass were measured in the lab while clothed but without shoes, jackets, or watches and jewelry during the first and fourth weeks of the Testing Phase to the nearest 0.1 cm and 0.1 kg using a Health-o-Meter beam scale (Continental Scale Corp., Bridgeview, IL)

**Table 2:** Weekly blood and urine collection and water pickup schedule during the 4-week Testing Phase.

Scheduled Event	Monday	Tuesday	Wednesday	Thursday	Friday	Saturday/Sunday
						
				
**Fingertip Blood**	*M1*	*M2*		*M3*		
				
					
**24-Hour Urine**	*M1*		*M2*			*M3*
					
						

**Bottled Water Pickup**	*AM Pickup*	*AM Pickup*	*AM Pickup*	*AM Pickup*	*AM Pickup*	*AM Pickup*

Note: M1-M3 refer to consecutive measurements #1 - #3 each week for both fingertip blood and 24-hour urine samples.

The daily lab visits also provided the opportunity for subjects to collect enough bottled water for their daily drinking needs. The placebo and AK water was provided to subjects in non-labeled water storage drums which had been filled in advance by the investigator. Subjects were individually assigned to draw their daily water needs from an assigned drum into color-coded non-labeled 1-liter plastic water storage bottles. Each subject was given as many 1-liter bottles as necessary to keep up with their daily water intake needs. Once emptied, subjects returned their 1-liter bottles to the lab the next day for refilling. The color-coding of these 1-liter bottles allowed the investigator to verify that subjects were drawing water from the correctly assigned water storage drum.

#### Fingertip Blood and 24-Hour Urine Collections

Subjects collected three 24-hour urine samples each week of the Testing Phase. A 24-hour sample was defined as the first urination following the morning's first void and all additional voids until and including the following morning's first void. Subjects were provided as many sterile 1-liter collection containers as needed for a 24-hour collection. Subjects were asked to store the urine containers during the day in their home refrigerator (approximately 4-8°C) until their return to the lab the next morning following the first void morning collection. Once at the lab, each subject's labeled containers were emptied into a sterile oversized mixing container and then measured for total urine volume using a one liter graduated cylinder to the hundredth of a liter. Prior to discarding the 24-hour sample, two 1.5-ml sterile sample vials were filled with urine and stored within a freezer (-18°C) until such time that all the samples could be thawed for the measurement of pH and osmolality. Each day's collection of urine samples were typically thawed within 48-72 hours following the initial freezer storage. Samples were allowed to thaw to room temperature (23°C) prior to the measurement of both pH and osmolality before returning to the freezer for storage.

Fingertip blood samples were collected using standard fingertip lancing and collection procedures into two 75 μl heparinized capillary tubes for an approximate collection volume of 75-100 μls. The contents of both capillary tubes were then emptied into a single 1.5-ml sample vial, labeled, and then stored in a lab refrigerator (4°C). The samples collected from each day were evaluated for both pH and osmolality 6-10 hours later that same day after warming to room temperature (23°C). The combination of the heparinized capillary tubes and refrigeration were sufficient to keep these small whole blood samples from coagulating prior to pH and osmolality measurements within the timeframe described.

#### 7-Day Physical Activity (PA) Assessment

Due to the time-intensive nature of the PA monitoring and diet diary analyses, the 7-day assessments were performed a total of three times over the 4-week Testing Phase instead of the entire four weeks. The first and third 7-day recordings of both types of data occurred Monday through Sunday for the entire pre- and post-treatment periods, respectively, while the second recordings occurred Wednesday through Tuesday in the middle of the treatment period.

Habitual free-living PA was evaluated using accelerometry-based activity monitors, or AMs, worn on the wrist using locking plastic wristbands (Wristband Specialty Products, Deerfield Beach, FL USA). Once locked onto the wrist with the wristband, the AM remained on the wrist for seven consecutive days until it was removed on the morning of the eighth day. A total of 40 AMs, all of which were calibrated by the manufacturer prior to testing, were randomly assigned to participants with participants using the same monitor for all three measurement periods. These data were used to determine the stability of the subjects' habitual free-living PA over the course of the Testing Phase.

The stability of dietary intake across the three measurement periods was evaluated on the basis of 7-day diet diaries. Subjects were provided a diet log book for each weekly assessment that included a sample one-day record, as well as figures illustrating common portion sizes. Once completed, the diet records were entered into Nutritionist Pro™ Diet Analysis software (Axxya Systems, Stafford, TX USA) for an evaluation of average daily macronutrient and micronutrient content, as well as average daily caloric intake. These data were also used to compute an estimate of the nutritionally-induced acid load on the body from the average intake of protein (Pro, g/day), phosphorus (P, mg/day), potassium (K, mg/day), calcium (Ca, mg/day), and magnesium (Mg, mg/day) by computing the potential renal acid load (PRAL) [12,13].

Finally, the diet diaries were also used to record self-report water consumption (SRWC, L/day) for the placebo and AK bottled waters provided by the lab to the nearest 0.1 liter. Bottled water consumption was recorded and analyzed separately from the diet diary analyses described above.

### Bottled Water Tested

The AK water consumed by the Experimental group (Akali^®^; Glacier Water Company, LLC; Auburn, WA USA) contains several naturally occurring trace minerals (silica, calcium, potassium, magnesium, selenium) in amounts ranging from 0.1-23.0 mg/L. When compared with public water sources, this mineral content is relatively high, though it is not uncommon for unfiltered glacier water melt. Indeed, AK water is one of several product lines from the same company which has sole bottling rights to the runoff from the Carbon Glacier on Mt. Rainier, WA. In addition to these natural minerals, AK water also contains an unknown amount of Alka-PlexLiquid™, a proprietary blend of mineral-based alkalizing agents said to be the active ingredient responsible for the water's unusually high pH of 10.0, as well as the previously reported enhanced rate of absorption and retention of water in the body [8].

The placebo water used for this study was Aquafina (PepsiCo Inc., Purchase, NY USA), a bottled water brand that is commonly available throughout the U.S. The bottlers of Aquafina use numerous public water sources across the U.S. and a trademarked purification process called HydRO-7™ that is said to remove all measureable traces of any particles that can influence water taste, including naturally occurring minerals. In fact, according to the Aquafina label, this purification process results in water that contains no significant minerals or electrolytes whatsoever. Thus, this particular bottled water is well suited to serve as a placebo for the present study.

Both placebo and AK bottled waters were shipped directly to the testing lab from their respective bottling facilities in previously unopened bottles. The contents of these bottles were emptied directly into the water storage drums used daily by the participating subjects as described previously. Using freshly opened bottles of water and the measurement procedures described below, the placebo and AK waters were measured at respective pH values of 7.0 and 10.0, while the osmolality for both waters was zero mOsm/kg. As a reference, a sample of distilled water had a pH of 7.0 and osmolality of zero mOsm/kg.

### Instrumentation

#### Osmolality and pH

Each urine and fingertip blood sample was evaluated for osmolality using the Model 3320 Micro-Osmometer (Advanced Instruments, Inc., Norwood, MA USA) to the nearest whole unit in mOsm/kg H_2_0. The osmometer was calibrated daily using standards of 50 to 2000 mOsm/kg as suggested by the manufacturer. In addition, this particular osmometer required only 20 μl to provide a valid measurement, which includes the measurements of whole blood, with an accuracy of ± 2 mOsm/kg within the 0-400 mOsm/kg range. The pH for the same urine and fingertip blood samples were determined using a Sentrol LanceFET pH Probe and Argus hand-held ISFET Ph meter (Topac Inc., Cohasset, MA USA). The pH probe had a range of 0-14 and a reported accuracy of ± 0.01 units while requiring only 20 μl for a valid measurement. The pH probe was calibrated prior to each run of measurements using two-point calibration routine with 4.0 and 7.0 pH standards provided by the manufacturer.

#### Physical Activity Monitors (AMs) and Data Processing Algorithm

The operating mechanism for the AM used for this study (Actical Monitor; Mini Mitter Company, Inc., Bend, OR USA) will be described briefly since it has been described in detail previously [14]. The AM is the size of a small wristwatch (2.8 × 2.7 × 1.0 cm^3^), light weight (0.017 kg), water resistant, utilizes a single "multidirectional" accelerometer to quantify motion, and has over five weeks of continuous data storage capacity using one-minute recording epochs. The raw AM data are stored in units of counts/min where a count is proportional to the magnitude and duration of accelerations during the user-specified epoch. When activity monitoring is complete, the raw AM data are downloaded to a computer using an external reader unit and a serial port connection as an ASCII formatted file. A custom Visual Basic (Version 6.0) computer program then transforms the minute-by-minute AM data into units of activity energy expenditure (AEE, kcals/kg/min) using a previously validated 2R algorithm [14] and post-processing methods [15,16] previously validated for wrist-worn monitoring in adults. For the present study, AEE was defined as the relative energy expenditure to perform a task above resting metabolism. Each subject's computed AEE data were then summarized into a time-based moderate-to-vigorous PA variable by summing the corresponding one-minute epochs greater than or equal to a moderate intensity cut point of 0.0310 kcals/kg/min [14]. This cut-point is the equivalent of the 3 MET cut point commonly used to define the lower boundary of moderate intensity in adults [17]. This processing routine was repeated with each ASCII formatted AM file to compute the 7-day average daily PA (mins/day) for each of the three periods within the Testing Phase.

### Statistical Analyses

Dependent variables for which there was only one value per measurement period (daily PA, SRWC, and all of the diet diary variables) were evaluated using two-factor multivariate repeated measures ANOVA and planned contrasts for post-hoc comparisons within the Control and Experimental group means. Thus, the analytical strategy was to identify changes in the dependent variables within the groups rather than between groups. All other dependent variables (blood and urine osmolality and pH, as well as 24-hour urine volume) were evaluated with a similar two-factor multivariate repeated measures ANOVA model, but Dunnett's test was used for post-hoc comparisons within the Control and Experimental group means. Dunnett's test compares the dependent variable means to a control, or reference condition. In the current study, no one measure could truly serve as a reference, so the mean of the pre-treatment values for each subject and each dependent variable was computed for use as this reference value. All ANOVA and post-hoc tests were performed at the 0.05 alpha level.

## Results

A total of 45 subjects were initially enrolled at the beginning of the Pilot Phase, but only 40 remained by the end the pre-treatment period of the Testing Phase. Four of the five subjects who dropped out did so of their own volition citing the time demand of the study, while the fifth subject dropped out of school and moved away from area. The remaining 40 subjects were evenly matched by gender and SRPA before assignment into the Control and Experimental groups. During third week of the Testing Phase, a sixth subject from the Control group dropped out due to unexpected out-of-town travel. Finally, the data from a seventh subject in the Experimental group was removed from the data pool prior to data analyses due to lack of consistent compliance with the study protocol. The demographic summary statistics for the remaining 38 subjects are provided in Table 3. Note that the Control and Experimental groups remained evenly balanced with 19 subjects each and nearly equal in numbers of male and female participants. While measures of body mass are shown only for the pre-treatment period (Table 3), these measures did not differ significantly from body mass measured during the post-treatment period.

**Table 3:** Summary of demographic data for study participants (Mean ± SD (Range)).

Group	Age (years)	Body Height (cms)	Body Mass (kg)	**†BMI (kg/m**^**2**^**)**	‡SRPA (hrs/wk)
**Control**					
**Women** **(n = 12)**	23 ± 3 (19 - 26)	169.1 ± 8.0 (153.3 - 185.3)	68.5 ± 7.3 (56.5 - 79.7)	23.9 ± 1.9 (21.5 - 28.6)	6.7 ± 4.6 (0 - 15.0)
**Men** **(n = 7)**	22 ± 1 (21 - 24)	182.2 ± 8.3 (175.3 - 199.6)	87.5 ± 7.5 (72.8 - 95.5)	26.4 ± 2.8 (22.7 - 31.1)	7.9 ± 2.7 (4.0 - 11.5)
**Experimental**					
**Women** **(n = 13)**	21 ± 2 (18 - 23)	168.3 ± 6.9 (161.0 - 182.2)	64.4 ± 8.8 (51.0 - 86.9)	22.7 ± 2.1 (19.3 - 26.5)	6.1 ±4.3 (0 - 15.0)
**Men** **(n = 6)**	24 ± 3 (21 - 28)	178.5 ± 5.6 (172.6 - 186.5)	80.8 ± 7.1 (70.8 - 91.2)	25.4 ± 2.8 (21.5 - 28.3)	6.8 ± 3.5 (2.8 - 11.3)

† BMI (Body mass index) = [(body mass, kg)/(body height, m)^2^]

‡ SRPA = Self-reported physical activity in hours per week.

### Daily PA, Water Consumption, and Diet Diaries

The Control and Experimental groups self-reported drinking similar amounts of the placebo and treatment water, respectively, provided by the study investigator (Table 4). For example, self-reported water consumption (SRWC) averaged 2.2-2.5 L/day for the Control group across all three test periods, while the Experimental group averaged 2.2-2.4 L/day. Daily PA, as determined with the wrist-worn physical activity monitors, was highest during the pre-treatment phase for both Control (Mean ± SE: 85 ± 8 mins/day) and Experimental (85 ± 6 mins/day) groups, and lowest for during the treatment phase (78 ± 8 and 70 ± 8 mins/day, respectively). None of the differences in SRWC or daily PA across test periods were significant within test groups (P > 0.20).

**Table 4:** Water consumption and physical activity for study participants reported as Mean ± SE (Range).

Group	Pre-Treatment Period		Treatment Period		Post-Treatment Period	
	†**SRWC (L/day)**	‡**Daily PA (mins/day)**	SRWC (L/day)	Daily PA (mins/day)	SRWC (L/day)	Daily PA (mins/day)
**Control**						
**Women** **(n = 12)**	2.5 ± 0.2 (1.7 - 4.8)	82 ± 9 (20 - 153)	2.4 ± 0.3 (1.2 - 5.0)	77 ± 12 (16 - 173)	2.2 ± 0.2 (1.3 - 4.7)	83 ± 12 (27 - 156)
**Men** **(n = 7)**	2.4 ± 0.4 (1.2 - 4.2)	92 ± 5 (78 - 109)	2.2 ± 0.4 (1.0 - 3.8)	82 ± 11 (60 - 135)	2.3 ± 0.5 (1.0 - 3.8)	74 ± 10 (45 - 106)
**Entire Group** **(n = 19)**	2.5 ± 0.2 (1.2 - 4.8)	85 ± 8 (20 - 153)	2.4 ± 0.3 (1.0 - 5.0)	78 ± 8 (16 - 173)	2.2 ± 0.3 (1.0 - 4.7)	80 ± 8 (27 - 156)

**Experimental**						
**Women** **(n = 13)**	2.0 ± 0.2 (1.0 - 4.1)	74 ± 9 (12 - 128)	1.9 ± 0.2 (1.0 - 4.0)	58 ± 6 (29 - 93)	1.7 ± 0.2 (1.0 - 3.0)	74 ± 10 (40 - 166)
**Men** **(n = 6)**	3.1 ± 0.2 (2.1 - 4.0)	105 ± 15 (41 - 170)	2.8 ± 0.5 (1.1 - 5.8)	91 ± 15 (15 - 127)	3.4 ± 0.4 (2.0 - 5.8)	92 ± 16 (47 - 145)
**Entire Group** **(n = 19)**	2.4 ± 0.2 (1.0 - 4.1)	85 ± 6 (12 - 170)	2.2 ± 0.2 (1.0 - 5.8)	70 ± 8 (15 - 127)	2.3 ± 0.2 (1.0 - 5.8)	81 ± 8 (40 - 166)

† SRWC = self-reported water consumption as recorded within food diaries.

‡ Daily PA = daily physical activity as determined with wrist-worn physical activity monitors.

Results from the diet diaries were also evaluated for changes in total caloric intake, macronutrient intake (protein, fat, and carbohydrate), mineral content (phosphorus, potassium, calcium, magnesium, sodium), as well as the number of food exchange equivalents for the consumption of fruits, vegetables, meat, starches, fat, and milk products. There were no significant changes for any these variables for either Control or Experimental groups across the three test periods (P > 0.10). In addition, the computation of average daily PRAL for the Control group did not change significantly between pre-treatment (20.5 ± 4.0 mEq/day), treatment (26.6 ± 6.4 mEq/day), and post-treatment (21.6 ± 5.0 mEq/day) phases (P = 0.29). Similarly, PRAL computations for the Experimental group did not change significantly across the same test periods (22.3 ± 5.6, 20.0 ± 5.0, and 32.2 ± 15.0 mEq/day, respectively) (P = 0.66).

### Blood and Urine Variables

**Figure 1:**
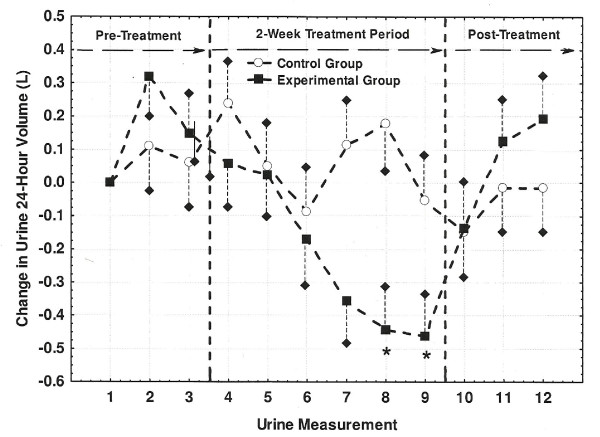
**Changes in 24-hour urine output (L/day) across the three study periods**. Changes are shown relative to the very first collection (i.e., urine measurement 1, or M1). Individual values were calculated as a difference between the measured value at each of the 12 measurements and the measured value at M1. Values marked with an asterisk (*) differed significantly from the M1 reference value of zero liters (P < 0.05). Short dashed lines represent one-side SE bars.

Daily urine output during the pre-treatment period averaged (Mean ± SE) 2.16 ± 0.24 and 2.67 ± 0.29 L/day for the Control and Experimental groups, respectively. Each subject's 24-hour urine output values were adjusted to change scores (i.e., 24-hour urine output minus output for first measurement) and where plotted in Figure 1. While urine output for the Control group did not change significantly over the course of the study, output for the Experimental group began decreasing by the sixth and seventh measurements (i.e., end of the first treatment week) with the last two treatment period collections being significantly lower (-0.44 to -0.46 L/day) than the reference value of zero L/day (P < 0.05).

Prior to the evaluation of osmolality and pH for the urine samples, both Control and Experimental groups were split into "low" and "high" subgroups using each group's respective median values for daily PA, SRWC, and average PRAL. These subgroups were used as a basis for reevaluating the urine measures since each of these variables can independently influence urine osmolality and pH. Summary statistics for PA, SRWC, and average PRAL for the resulting subgroups are provided in Table 5. A complete summary of urine osmolality results are provided in Tables 6 and 7 for Control and Experimental groups, respectively. There were no significant changes in urine osmolality for the Control group over the entire Testing Phase, regardless of whether the entire group or subgroups were evaluated. Urine osmolality for urine samples collected in the second week of the treatment period for the Experimental group, however, were significantly higher than the pre-treatment reference value. The subgroup analyses also indicated that urine osmolality tended to be significantly higher at the end of the treatment period for Experimental subjects within the "high" daily PA, "low" SRWC, and "high" PRAL subgroups. Tables 8 and 9 show that the trends for changes in urine pH paralleled those discussed for urine osmolality. Specifically, there were no significant changes in urine pH across all measurements for the Control group which includes the daily PA, SRWC, and PRAL subgroup analyses (Table 8). In contrast, when considering the Experimental group urine measures (Table 9), pH increased progressively and significantly throughout the treatment period by approximately 0.3 to 0.8 units. This same trend was evident throughout the "low" and "high" Experimental subgroup analyses as well with the largest pH increases (+0.5 to +1.2 units) observed for the "high" daily PA, "high" SRWC, and "high" PRAL subgroups. Interestingly, observed changes in daily urine output, osmolality, and pH for the Experimental group all returned to pre-treatment levels during the post-treatment period.

**Table 5:** Summary statistics of sub-group analysis variables reported as Mean ± SD (Range).

Grouping Variables	Control Group (n = 19)		Experimental Group (n = 19)	
	"Low" (n = 9)	"High" (n = 10)	"Low" (n = 9)	"High" (n = 10)
†**Daily PA (mins/day)**	41.2 ± 14.7 (15.0 - 63.0)	96.6 ± 19.9 (68.0 - 127.0)	51.3 ± SD (16.0 - 73.0)	102.7 ± 32.6 (75.0 - 173.0)
‡**SRWC (L/day)**	1.4 ± 0.3 (1.0 - 1.9)	3.1 ± 1.1 (2.0 - 5.6)	1.4 ± 0.23 (1.0 - 1.7)	2.95 ± 0.84 (1.8 - 4.7)
§**PRAL (mg/day)**	5.72 ± 9.40 (-8.30 - 23.9)	45.30 ± 25.85 (24.60 - 114.90)	3.28 ± 11.8 (-22.2 - 15.0)	35.05 ± 17.3 (18.4 - 74.0)

† SRWC = self-reported water consumption as recorded within food diaries.

‡ Daily PA = daily physical activity as determined with wrist-worn physical activity monitors.

§ PRAL = potential renal acid load as computed from diet diary evaluations.

**Table 6:** Urine Osmolality for the Control group with daily PA, SRWC, and PRAL subgroup analyses (Mean (SE)).

Control Condition	Pre-Treatment Period			Treatment Period						Post-Treatment Period		
	M1	M2	M3	M4	M5	M6	M7	M8	M9	M10	M11	M12
**All Subjects**	495	424	466	450	439	470	419	448	430	480	488	425
**(n = 19)**	(52)	(42)	(54)	(51)	(55)	(42)	(41)	(42)	(50)	(54)	(47)	(43)

**Low PA** **(n = 9)**	509 (64)	478 (67)	483 (69)	512 (76)	515 (70)	418 (76)	461 (80)	465 (78)	445 (81)	493 (77)	468 (79)	479 (50)
**High PA** **(n = 10)**	483 (66)	375 (56)	451 (57)	394 (40)	370 (41)	516 (60)	382 (36)	370 (35)	416 (50)	461 (68)	506 (57)	467 (68)

**Low SRWC** **(n = 9)**	538 (66)	499 (55)	538 (69)	502 (60)	469 (67)	506 (71)	426 (37)	430 (36)	470 (67)	515 (61)	483 (54)	433 (52)
**High SRWC** **(n = 10)**	456 (69)	356 (56)	402 (72)	403 (69)	412 (70)	437 (50)	413 (72)	410 (70)	394 (58)	446 (69)	493 (77)	419 (69)

**Low PRAL** **(n = 9)**	466 (64)	444 (72)	495 (69)	452 (75)	457 (76)	455 (77)	398 (44)	410 (44)	441 780)	493 (74)	468 (63)	380 (59)
**High PRAL** **(n = 10)**	521 (66)	406 (49)	440 (68)	448 (72)	423 (72)	480 (60)	438 (69)	435 (60)	442 (80)	466 (69)	506 (71)	466 (62)

Note: There were a total of twelve 24-hour urine collections labeled in the table as M1-M12, respectively. Mean osmolality values were compared directly with respective mean Pre-Treatment reference value which were averages of all M1-M3 values within the condition and subject group being evaluated. These Pre-Treatment reference values were as follows: 462 (all Control subjects), 490 (low PA), 436 (high PA), 525 (low SRWC), 405 (high SRWC), 468 (low PRAL), and 456 mOsm/kg (high PRAL). There were no significant differences detected for any of the evaluations.

**Table 7:** Urine Osmolality for the Experimental group with daily PA, SRWC, and PRAL subgroup analyses (Mean (SE)).

Experimental Condition	Pre-Treatment Period			Treatment Period						Post-Treatment Period		
	M1	M2	M3	M4	M5	M6	M7	M8	M9	M10	M11	M12
**All Subjects**	373	367	387	375	343	396	**† **435	**† **440	**† **445	376	358	360
**(n = 19)**	(28)	(39)	(47)	(32)	(40)	(42)	(41)	(44)	(40)	(38)	(31)	(35)

**Low PA** **(n = 9)**	372 (45)	390 (68)	409 (73)	403 (52)	368 (79)	379 (80)	444 (87)	451 (87)	417 (82)	426 (64)	383 (49)	420 (70)
**High PA** **(n = 10)**	374 (36)	346 (45)	368 (63)	350 (41)	330 (56)	412 (51)	427 (48)	430 (50)	**† **473 (45)	330 (42)	335 (40)	340 (45)

**Low SRWC** **(n = 9)**	418 (39)	477 (58)	505 (79)	467 (41)	460 (43)	504 (47)	**† **574 (46)	**† **581 (45)	**† **562 (46)	441 (59)	414 (41)	480 (70)
**High SRWC** **(n = 10)**	333 (37)	268 (28)	281 (28)	292 (31)	238 (36)	299 (29)	310 (42)	315 (43)	332 (45)	318 (44)	308 (41)	354 (36)

**Low PRAL** **(n = 9)**	355 (44)	342 (61)	450 (65)	343 (38)	336 (40)	362 (45)	412 (49)	419 (50)	376 (50)	345 (46)	351 (49)	413 (65)
**High PRAL** **(n = 10)**	390 (36)	389 (51)	331 (46)	404 (51)	349 (42)	427 (44)	456 (48)	**† **460 (45)	**† **470 (45)	404 (61)	365 (41)	4141 (39)

Note: There were a total of twelve 24-hour urine collections labeled in the table as M1-M12, respectively.

**† **Mean osmolality value differed significantly (P < 0.05) from respective mean Pre-Treatment reference value which was an average of all M1-M3 values within the condition and subject group being evaluated. These Pre-Treatment reference values were as follows: 376 (all Experimental subjects), 390 (low PA), 363 (high PA), 467 (low SRWC), 294 (high SRWC), 382 (low PRAL), and 370 mOsm/kg (high PRAL).

**Table 8:** Urine pH for the Control group with daily PA, SRWC, and PRAL subgroup analyses (Mean (SE)).

Control Condition	Pre-Treatment Period			Treatment Period						Post-Treatment Period		
	M1	M2	M3	M4	M5	M6	M7	M8	M9	M10	M11	M12
**All Subjects**	6.01	6.11	6.13	6.13	6.20	6.15	6.01	6.01	6.00	6.08	5.86	6.20
**(n = 19)**	(0.11)	(0.09)	(0.08)	(0.10)	(0.11)	(0.06)	(0.07)	(0.07)	(0.08)	(0.09)	(0.08)	0.08)

**Low PA** **(n = 9)**	5.95 (0.21)	5.93 (0.11)	6.00 (0.14)	6.07 (0.16)	6.12 (0.17)	6.11 (0.09)	5.86 (0.07)	5.86 (0.07)	5.91 (0.11)	6.02 (0.14)	5.99 (0.12)	6.11 (0.12)
**High PA** **(n = 10)**	6.05 (0.11)	6.20 (0.10)	6.24 (0.10)	6.19 (0.13)	6.36 (0.12)	6.19 (0.09)	6.14 (0.12)	6.14 (0.12)	6.05 (0.12)	6.14 (0.12)	6.02 (0.08)	6.28 (0.11)

**Low SRWC** **(n = 9)**	6.21 (0.18)	6.28 (0.13)	6.17 (0.17)	6.13 (0.15)	6.17 (0.13)	6.29 (0.14)	5.85 (0.14)	5.85 (0.14)	5.99 (0.12)	6.25 (0.12)	6.16 (0.16)	6.37 (0.14)
**High SRWC** **(n = 10)**	6.30 (0.18)	6.15 (0.10)	6.14 (0.09)	6.18 (0.14)	6.31 (0.15)	6.18 (0.14)	6.25 (0.15)	6.25 (0.15)	6.19 (0.13)	6.15 (0.11)	5.94 (0.13)	6.10 (0.11)

**Low PRAL** **(n = 9)**	6.06 (0.22)	6.11 (0.16)	6.22 (0.15)	6.22 (0.17)	6.23 (0.17)	6.23 (0.11)	5.92 (0.11)	5.92 (0.11)	5.92 (0.13)	5.98 (0.16)	5.87 (0.15)	6.16 (0.14)
**High PRAL** **(n = 10)**	5.96 (0.10)	6.11 (0.09)	6.04 (0.09)	6.06 (0.11)	6.36 (0.36)	6.08 (0.07)	6.08 (0.10)	6.08 (0.10)	6.04 (0.10)	6.18 (0.08)	5.86 (0.09)	6.24 (0.09)

Note: There were a total of twelve 24-hour urine collections labeled in the table as M1-M12, respectively. Mean pH values were compared directly with respective mean Pre-Treatment reference value which were averages of all M1-M3 values within the condition and subject group being evaluated. These Pre-Treatment reference values were as follows: 6.08 (all Control subjects), 5.96 (low PA), 6.16 (high PA), 6.22 (low SRWC), 6.20 (high SRWC), 6.13 (low PRAL), and 6.04 (high PRAL).

**Table 9:** Urine pH for the Experimental group with daily PA, SRWC, and PRAL subgroup analyses (Mean (SE)).

Experimental Condition	Pre-Treatment Period			Treatment Period						Post-Treatment Period		
	M1	M2	M3	M4	M5	M6	M7	M8	M9	M10	M11	M12
**All Subjects**	6.28	6.20	6.22	6.25	**† **6.51	**† **6.57	**† **7.00	**† **7.00	**† **7.07	6.23	6.17	6.21
**(n = 19)**	(0.11)	(0.11)	(0.10)	(0.10)	(0.09)	(0.10)	(0.12)	(0.11)	(0.08)	(0.07)	(0.10)	(0.09)

**Low PA** **(n = 9)**	6.34 (0.16)	6.40 (0.18)	6.32 (0.12)	6.32 (0.12)	6.54 (0.13)	6.63 (0.12)	**† **6.88 (0.12)	**† **6.89 (0.13)	**† **6.94 (0.08)	6.34 (0.11)	6.24 (0.17)	6.33 (0.17)
**High PA** **(n = 10)**	6.23 (0.15)	6.02 (0.12)	6.11 (0.14)	6.04 (0.09)	6.48 (0.11)	**† **6.67 (0.13)	**† **7.15 (0.13)	**† **7.12 (0.13)	**† **7.10 (0.13)	6.13 (0.12)	6.11 (0.12)	6.11 (0.12)

**Low SRWC** **(n = 9)**	6.17 (0.09)	6.26 (0.14)	6.33 (0.09)	6.21 (0.10)	6.30 (0.08)	6.29 (0.12)	6.34 (0.11)	6.54 (0.11)	**† **6.60 (0.11)	6.16 (0.11)	6.11 (0.09)	6.09 (0.08)
**High SRWC** **(n = 10)**	5.91 (0.16)	5.96 (0.18)	6.00 (0.16)	6.29 (0.17)	**† **6.57 (0.17)	**† **6.78 (0.11)	**† **7.21 (0.12)	**† **7.14 (0.14)	**† **7.25 (0.08)	6.07 (0.16)	5.88 (0.15)	6.27 (0.12)

**Low PRAL** **(n = 9)**	6.56 (0.15)	6.40 (0.16)	6.46 (0.12)	6.41 (0.13)	6.50 (0.11)	6.50 (0.14)	**† **6.79 (0.20)	**† **6.88 (0.20)	**† **6.89 (0.14)	6.40 (0.10)	6.32 (0.15)	6.37 (0.14)
**High PRAL** **(n = 10)**	6.04 (0.11)	6.02 (0.13)	5.99 (0.15)	6.19 (0.15)	**† **6.63 (0.14)	**† **6.65 (0.14)	**† **7.15 (0.13)	**† **7.18 (0.13)	**† **7.24 (0.07)	6.07 (0.12)	6.04 (0.12)	6.07 (0.08)

Note: There were a total of twelve 24-hour urine collections labeled in the table as M1-M12, respectively.

**† **Mean pH value differed significantly (P < 0.05) from respective mean Pre-Treatment reference value which was an average of all M1-M3 values within the condition and subject group being evaluated. These Pre-Treatment reference values were as follows: 6.23 (all Experimental subjects), 6.35 (low PA), 6.12 (high PA), 6.33 (low SRWC), 5.96 (high SRWC), 6.47 (low PRAL), and 6.02 (high PRAL).

**Figure 2:**
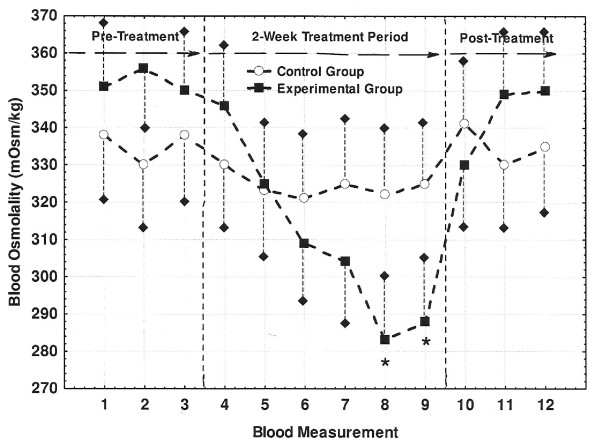
**Changes in fingertip blood osmolality across the three study periods**. Blood osmolality values correspond each of twelve (i.e., M1-M12) fingertip collections. Values marked with an asterisk (*) differed significantly from the M1 reference values of 335 and 352 mOsm/kg for the Control and Experimental groups, respectively (P < 0.05). Short dashed lines represent one-side SE bars.

**Figure 3:**
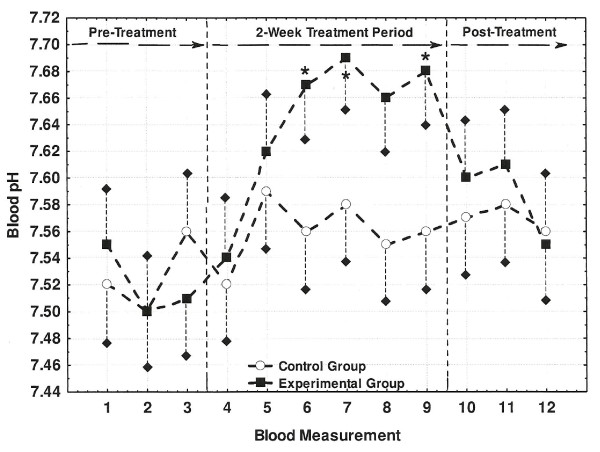
**Changes in fingertip blood pH across the three study periods**. Blood pH values correspond each of twelve (i.e., M1-M12) fingertip collections. Values marked with an asterisk (*) differed significantly from the M1 reference values of 7.53 and 7.52 for the Control and Experimental groups, respectively (P < 0.05). Short dashed lines represent one-side SE bars.

Fingertip blood osmolality and pH measurements for both Control and Experimental groups are shown in Figures 2 and 3, respectively. While blood osmolality showed no significant changes for Control group, blood osmolality progressively decreased from the start to the end of the treatment period with the last two measures significantly lower than the pre-treatment reference value. The Control group's blood pH also showed no significant changes while the Experimental group's blood increased significantly by 0.15-0.17 units by the second week of the treatment period. Similar to the observations described for the urine measures, blood osmolality and pH both returned to pre-treatment levels during the post-treatment period.

## Discussion

This study was designed to evaluate the influence of mineralized alkaline bottled water (i.e., AK water) on markers of both acid-base balance and hydration status. In particular, these measurements were performed under free-living conditions, meaning that there was no purposeful attempt to control individual differences in daily PA, dietary intake, or even daily water consumption. As such, the design of this study should allow for the results to be more generalizable to the habitual consumption of bottled water than would results from a laboratory controlled study.

### Influence on Acid-Base Balance

When compared with the consumption of the placebo bottled water, habitual consumption of AK water in the present study was associated with an increase in both urine (Table 7) and blood (Figure 3) pH while measures of both daily PA (Table 4) and dietary composition remained stabile. Previous research by Welch et al. [11] demonstrated that urinary pH from 24-hour collection samples could function as an effective surrogate marker for changes in acid-base balance when evaluating differences in dietary intake. König et al [10] used this information as a premise for determining that consumption of a mineral-rich supplement significantly increased both urine (5.94 to 6.57) and blood pH (7.40 to 7.41). Similarly, Berardi et al. [9] showed that urinary pH increased from 6.07 to 6.21 and 6.27 following one and two weeks of ingestion, respectively, of a plant-based supplement. The observations from these studies [9,10] are consistent with the changes in urine (6.23 to 7.07) and blood pH (7.52 to 7.69) observed by the present study for the Experimental group. Thus, the habitual consumption of AK water under free-living conditions had a similar influence on urinary and blood pH as has been shown to occur with nutrition supplements specifically designed to impact the body's acid-base balance.

The above observations, however, are not without limitations as the onset and magnitude of the urine alkalization within the Experimental group was influenced by daily PA, SRWC, and computed dietary PRAL (Table 9). Specifically, urine pH tended to increase sooner within the treatment period and to a higher pH level for those who habitually engaged in more physical activity, self-reported drinking more AK water, as well as those who regularly reported higher nutritionally-induced acid loads (Table 9). Thus, the actual impact of consuming the AK water's mineral-based alkalizing agents on urine pH may be dose dependent. This observation would certainly explain the differences in urinary pH between "low" and "high" levels of AK water consumption and daily PA, but a study that precisely controls AK water intake is needed to support the speculation of a dose-response relationship.

It is interesting to note that the blood pH values reported for this study are somewhat higher than the 7.35-7.45 range typically ascribed as the ideal range for blood pH. It is likely that the measurement procedures used (i.e., fingertip samples collected in heparinized capillary tubes and refrigerator stored for 6-10 hrs) allowed the samples to slightly increase pH prior to the actual measurement of pH. However, since this effect would have been the same for both Control and Experimental subjects, it is presumed that this effect was similar for all samples. Thus, while the blood pH values are slightly elevated for both Control and Experimental groups, the significant change in blood pH demonstrated by the Experimental group is likely a real effect of consuming AK water.

### Influence on Hydration Status

Consumption of AK water following a dehydrating bout of cycling exercise has previously been shown to rehydrate cyclists faster and more completely than the consumption of placebo bottled water (i.e., Aquafina) [8]. Following the consumption of AK water, the cyclists demonstrated less total urine output, their urine was more concentrated (higher specific gravity), and total blood protein concentration was lower, all of which are expected observations for improved hydration status [8]. Even though the present study was performed under free-living conditions, the Experimental group demonstrated an increased urine concentration (osmolality; Table 7), a decreased total urine output (Figure 1), as well as a decreased blood osmolality (Figure 2) by the end of the treatment period. These changes suggest that while SRWC was relatively stabile across measurement periods (Table 4), a relatively greater proportion of the AK water consumed during the treatment phase was being retained within the cardiovascular system. Indeed, the cyclist hydration study described above [8] reported that water retention at the end of a 3-hour recovery period was 79.2 ± 3.9% when subjects drank AK water versus 62.5 ± 5.4% when drinking the placebo (P < 0.05). Thus, the present study has shown that the habitual consumption of mineralized bottled water can actually improve indicators of hydration status over non-mineralized bottled water under free-living conditions that is consistent with lab-controlled study results.

Similar to what was described for changes in acid-base balance above, however, the onset of these observations did not begin with the immediate consumption of AK water. In fact, changes in total urine output, urine osmolality, and blood osmolality did not appear to begin changing until the end of the first week of consuming AK water, with significant changes always occurring at the end of the second week of consumption. Unfortunately, the present study was designed to observe possible changes in acid-base balance and hydration status rather than decipher mechanistic causes. However, it is possible to speculate on some contributing causes given that the AK water manufacturer lists only three major naturally occurring minerals on the bottle label (Calcium at 2.8 mg/L, Silica at 16.0 mg/L, and Potassium at 23.0 mg/L) as well as the proprietary blend of mineral-based alkalizing supplement called Alka-PlexLiquid™. According to the manufacturer, Alka-PlexLiquid™ is a freely dissolvable form of a patented blend of mineral-based alkalizing ingredients called Alka-Plex™ granules. These granules are packaged in tablet form and sold as one of several types of nutrition and sports performance supplements and has been granted New Dietary Ingredient (NDI) recognition by the Food and Drug Administration (FDA). According to the Alka-Plex™ product labels, as well as literature made available by the manufacturer, Alka-Plex™-based products contain a considerable amount of calcium carbonate, potassium hydroxide, magnesium hydroxide, and potassium chloride. Since all of these compounds will freely disassociate in a water solution, there will be an unusually high concentration of the same minerals already present in AK's glacier water (calcium, potassium, magnesium), as well as the alkaline half of these compounds (e.g., hydroxide ion, or OH^-^, from potassium hydroxide). Though the exact amounts of these Alka-Plex™-based compounds within the Alka-PlexLiquid™ formula are not known, these compounds are likely the driving force behind the observations in the present study. It is possible, for example, that the continual presence of a dietary alkalizing agent absorbed directly into the blood could eventually shift blood pH upward while having the greatest impact on urinary pH for those consuming relatively acidic diets. In fact, urinary pH was influenced the most for those in the Experimental group with the highest PRAL values (Table 9). It is also possible that the influx of additional minerals absorbed into the blood from the AK water contributed to a greater retention of water within the cardiovascular system. This hypothesis could explain why urine output for the Experimental group increased during the post-treatment period following the shift from consuming AK water to the placebo water. Clearly, to understand the cause behind the observations from the present study, more work on tracking concentration changes of these key minerals in both the blood and urine should occur.

### Study Implications

The results from this study suggest that the regular consumption of mineral-rich bottled water with the Alka-PlexLiquid™ supplement can have measureable influences on markers for acid-base balance and hydration status when consumed under free-living conditions. Since most studies evaluating nutritional influences on acid-base status are either large-scale epidemiological studies [11], or studies where dietary or supplement intake is tightly controlled [10], the present study is relatively unique. The self-regulation of water consumption by subjects in the present study, however, also make it somewhat more difficult to definitively state how much AK water should be consumed to realize similar observations. Regardless, the present study results suggest that the influence of drinking AK water requires either an exposure period (i.e., ≥1 week) or a minimal volume of AK water consumption before the effects can be detected significantly in the blood and urine. While the minimal volume consumed to detect changes in pH or hydration status is likely to be influenced by diet and daily PA, an estimate can be computed based upon the results discussed thus far. For example, if it is assumed that AK water is being consumed at an average rate of 2.3 L/day (an average of rates from Table 4), and that at least a week of regular consumption is required for hydration and/or pH influence is detectable, then the minimal consumption required under free-living conditions is approximately 16 L (i.e., 2.3 L/day × 7 days = 16.1 L) in young healthy adults. However, the "high" SRWC Experimental subgroup (SRWC = 3.0 L/day; Table 4) showed significantly increased urine pH by only the second urine measurement during the treatment period, which translates to a minimal consumption rate of approximately 9 L over three days rather than 16 L over seven days. These computations are for illustration purposes to highlight the fact that the "dose" of AK water consumption needed to elicit a particular blood or urine "response" should be evaluated more precisely in future studies.

Low-grade metabolic acidosis is generally considered to be a predisposing risk factor for the development of several chronic conditions [1-4]. While it has been suggested that the alkalizing influence of dietary interventions and supplements can be an important countering influence [7], the present study was not designed to determine whether the consumption of AK water could improve these disease conditions or not. However, given that the influences on blood and urine pH were consistent with the hypothesized changes, that the changes reversed during the post-treatment period, and that the Control group showed no changes over the same time period, it is reasonable to suggest that the consumption of AK water could be utilized in a clinical trial where those with a specific chronic disease or condition are targeted.

## Conclusions

The consumption of the mineral-rich bottled water with the Alka-PlexLiquid™ supplement (Akali^®^, or AK water) was associated with improved acid-base balance (i.e., an alkalization of the blood and urine) and hydration status when consumed under free-living conditions. In contrast, subjects who consumed the placebo bottled water showed no changes over the same period of time. These results indicate that the habitual consumption of AK water may be a valuable nutritional vector for influencing both acid-base balance and hydration status in healthy adults.

## Competing interests

The author declares that they have no competing interests.

## Authors' contributions

The author of this study is solely responsible for the study design, subject recruitment and health screening, data analysis, and manuscript preparation.
